# Knowledge structure and evolution of masked mycotoxin research in Sub-Saharan Africa: A systematic review and network analysis approach

**DOI:** 10.1016/j.toxcx.2025.100232

**Published:** 2025-09-17

**Authors:** Chimwemwe Chilenga, William Kasapila, Kingsley Masamba, Tinnah Manani, Victor Munkhuwa, Brown Ndhlovu, Kennedy Machira

**Affiliations:** a Department of Food Science and Technology, Lilongwe University of Agriculture and Natural Resources (LUANAR), Bunda College Campus, P.O. Box 219, Lilongwe, Malawi; b Department of Nutrition, HIV and AIDS, Ministry of Health, P/Bag B401, Lilongwe, Malawi; c Browns Consulting Company, P.O. Box 374, Rumphi, Malawi; d Ministry of Health, Lilongwe District Hospital, P.O. Box 1274, Lilongwe, Malawi; e Department of Agricultural Economics, Africa Center of Excellence in Agriculture Policy Analysis (APA), LUANAR, P.O. Box 219, Lilongwe, Malawi

## Abstract

Masked mycotoxins are modified forms of mycotoxins that escape conventional detection, posing underexplored risks to food safety. Despite their potential public health risks, research on these compounds remains limited in Sub-Saharan Africa (SSA). This study systematically reviewed 22 publications, analyzing research trends, geographic focus, and knowledge gaps using network analysis to assess the evolution and structure of masked mycotoxin research in SSA. Studies began in 2013, grew slowly with one study per year from 2014 to 2017, and modestly increased to 2–4 studies annually between 2018 and 2024. Geographically, research efforts are concentrated in a few countries, particularly Nigeria (47.6 % of publications), with Ethiopia, South Africa, Kenya, and Namibia contributing sporadically. The findings reveal that only 13.6 % of the studies had masked, modified, emerging, or hidden mycotoxins as part of the primary focus of the study objectives, while the majority included them as ancillary findings. The most prevalent masked mycotoxins identified are derivatives of aflatoxins and fumonisins, which pose significant risks to food safety and public health. Emerging challenges include the limited detection capabilities and weak regulatory frameworks on masked mycotoxins, with many studies failing to capture the full extent of their impact. Notably, no systematic reviews were found to focus exclusively on masked mycotoxins, indicating a major research gap. The field remains fragmented and underdeveloped, with significant limitations in analytical capacity and geographic scope. Addressing these gaps requires enhanced regional collaboration, increased funding for targeted research, and the integration of masked mycotoxin monitoring into national food safety policies.

## Highlights

• Few studies focused on masked mycotoxins despite known health risks.
• Only 13.6 % of studies had masked mycotoxins as a primary research focus.
• Nigeria accounted for 45.5 % of studies; other countries showed limited output.
• Detection, regulation, and research coordination gaps persist across SSA.

## Graphical abstract

**Figure:**
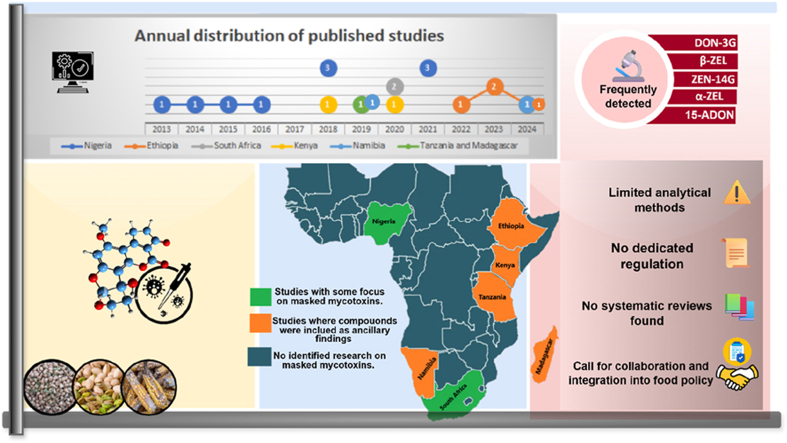

## 1 Introduction

Mycotoxins are toxic secondary metabolites produced by fungi such as *Aspergillus*, *Fusarium*, and *Penicillium*. Additionally, other species like *Alternaria*, *Claviceps*, and *Stachybotrys* are known to produce mycotoxins, though to a lesser extent (Omotayo et al., 2019; Sserumaga et al., 2021).*Fusarium* species produce mycotoxins like fumonisins (FB1, FB2, FB3, and FB4), zearalenones, fusaric acid, fusarin (C, D, and E), deoxynivalenol (DON), nivalenol, and T-2 toxin. Meanwhile, *Aspergillus* species synthesize aflatoxins (AFB1, AFB2, AFG1, and AFG2), ochratoxin, gliotoxins, and cyclopiazonic acid, while *Penicillium* produces ochratoxin, patulin, citrinin, and penicillic acid (Chilenga et al., 2024; Kebede et al., 2020; Magamba et al., 2017; Matumba, 2014) Collectively, these are known as free mycotoxins. These mycotoxins are invisible to the naked eye and tasteless, yet they contaminate essential crops such as maize, wheat, rice, groundnuts, and soybeans, presenting substantial risks to food safety and human health globally (Alshannaq and Yu, 2017; Fernández-Cruz et al., 2010; Milicevic et al., 2015)**.**

**Fig. 1:**
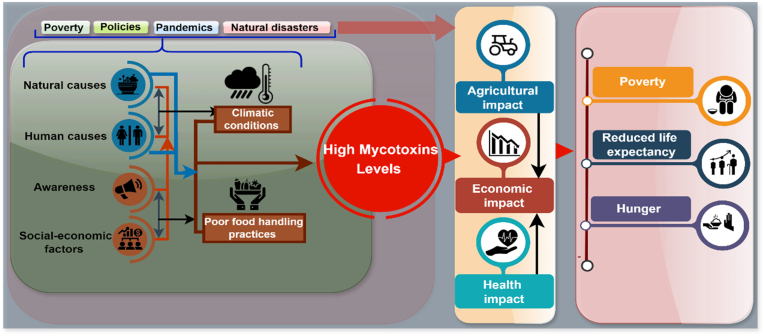
Illustration of the key factors contributing to elevated mycotoxin levels and their associated impacts.

Sub-Saharan Africa experiences high levels of mycotoxin contamination, owing to a combination of factors, including a climate favorable to fungal growth, socioeconomic challenges such as poverty, inadequate policies, pandemics, natural disasters, and suboptimal agricultural and food handling practices (Chilenga et al., 2025; Misihairabgwi et al., 2019; Nji et al., 2022; Nleya et al., 2018b). The resultant high levels of mycotoxins have been linked to reduced agricultural productivity, increased incidences of liver cancer and stunted growth, and substantial economic losses due to rejected exports and compromised food security (Benkerroum, 2020; Gong et al., 2016; Xu et al., 2018). Children and pregnant women are especially vulnerable due to their developing immune systems and the potential for transplacental transfer and exposure through breast milk (Adaku Chilaka and Mally, 2020; Brevik et al., 2024). Fig. 1 below provides a detailed illustration of the factors contributing to elevated mycotoxin levels and their impacts.

Mitigating their impact is particularly challenging because these toxins are chemically stable, heat-resistant, and can survive typical food processing methods. In developing countries, efforts to address mycotoxins often concentrate on aflatoxins, leaving other harmful mycotoxins unregulated and overlooked, resulting in significant gaps in food safety management systems (Paca, 2020).

While free mycotoxins remain the primary concern, an emerging and often underestimated risk has arisen in the form of masked mycotoxins. Masked mycotoxins are derivatives of parent fungal toxins that have undergone chemical modifications such as conjugation with sugars, amino acids, or sulphates primarily through plant metabolism or microbial contamination (Berthiller et al., 2013; Goryacheva and De Saeger, 2012; Gratz, 2017). This biochemical transformation renders them “masked” because they are undetectable by conventional analytical methods designed to identify free mycotoxins (Rychlik et al., 2014). The concern with masked mycotoxins lies in their ability to be hydrolyzed back into their toxic parent compounds during digestion or food processing, thereby representing a hidden threat to food safety and public health (Berthiller et al., 2013; Gratz, 2017). This risk is particularly acute in Sub-Saharan Africa, where staple crops are frequently contaminated and food safety systems are still evolving (Akello et al., 2021; Getahun et al., 2023; Nleya et al., 2018a; Ssepuuya et al., 2018).

Unlike free mycotoxins, such as aflatoxins, which are well-regulated with established legislative frameworks and clear maximum permissible levels in many countries, masked mycotoxins pose greater challenges for detection and quantification due to their altered chemical structures and contamination (Berthiller et al., 2013; Goryacheva and De Saeger, 2012; Gratz, 2017). Consequently, food products may appear safe and meet certification standards despite harboring hidden risks. Although masked mycotoxins are generally less toxic in their conjugated form, their conversion back into parent toxins, such as DON-3-glucoside to DON, or ZEN-14-sulfate to ZEN, by intestinal microbiota, presents significant health hazards, including liver cancer and immune system impairment (Kovalsky et al., 2016; Chilaka et al., 2022). This dual threat of invisibility and reactivation complicates mycotoxin risk management, particularly in regions still addressing the fundamental challenges of free mycotoxins. Moreover, these toxins remain poorly studied and difficult to monitor, despite posing serious threats to both human and animal health. With global trade expansion and climate change favoring fungal proliferation, the prevalence and impact of masked mycotoxins are expected to increase, further complicating efforts to ensure safe food systems (Hove et al., 2016; Motloung et al., 2018; Tebele et al., 2020a; Warth et al., 2012).

In addition to free and masked mycotoxins, emerging mycotoxins add a new dimension to global food safety challenges. Emerging mycotoxins are newly identified or previously underappreciated toxins arising from novel fungal species, environmental changes, or shifts in agricultural practices. These toxins, such as enniatins, beauvericin, and fusaproliferin, produced by *Fusarium* species, exhibit diverse biological activities, including cytotoxicity and immunomodulation, which raises concerns about their health impact (Ekwomadu et al., 2019; Falavigna et al., 2012; Omotayo et al., 2019; Zhang et al., 2020). Climate change and globalization have accelerated the spread and significance of these emerging mycotoxins, increasing exposure risks worldwide (Kostelanska et al., 2011; Kozieł et al., 2021; Lancova et al., 2008; Lattanzio et al., 2012; Olopade et al., 2021). The growing threat of masked mycotoxins in Sub-Saharan Africa necessitates a thorough understanding of current knowledge and research trends. Despite the severe implications for human and animal health, the literature on masked mycotoxins remains fragmented and insufficient, especially given the region's heavy agricultural dependence and vulnerability to contamination. This systematic literature review and network analysis aim to address this knowledge gap by mapping the state of research on masked mycotoxins in Sub-Saharan Africa. Specifically, this study will:

1. Map the current state of knowledge on masked mycotoxins in Sub-Saharan Africa, highlighting key findings, trends, and patterns.
2. Analyze the evolution of research on masked mycotoxins over time, identifying emerging themes and knowledge gaps.
3. Identify critical research needs to inform evidence-based policies and interventions.

The findings are expected to contribute significantly to understanding masked, hidden, modified, or emerging mycotoxins, guiding researchers, policymakers, and stakeholders in agriculture and public health sectors. By recognizing limitations and research scarcity across Sub-Saharan African countries and crops, this review aims to stimulate further research and collaborative efforts to address this urgent food safety concern.

## 2 Methods and materials

This study utilized a systematic review and network analysis method to investigate the knowledge structure and evolution of masked mycotoxins research in Sub-Saharan Africa. To ensure a rigorous and comprehensive literature search while maintaining data integrity, the review adhered to the methodological standards and procedural framework established by the Preferred Reporting Items for Systematic Reviews and Meta-Analyses (PRISMA) guidelines (Page et al., 2021). Dimensions AI served as the primary database for this review due to its extensive coverage of publications, advanced search capabilities, daily updates, and detailed metadata, which facilitated the precise retrieval of relevant studies and enabled comprehensive network analysis. Key search terms included “modified mycotoxin,” “masked mycotoxin,” “hidden mycotoxin,” and “emerging mycotoxin.” Data from various Excel files retrieved from the database were merged, screened, and refined. The inclusion criteria encompassed articles focused on mycotoxins in Sub-Saharan Africa and addressed masked, hidden, modified, or emerging mycotoxins. For an article to be included, the terms “masked mycotoxin,” “modified mycotoxin,” “hidden mycotoxin,” or “emerging mycotoxin” needed to appear in either the title or abstract. Research articles published in English from 2000 to 2025 were included to ensure a comprehensive and robust evidence base for this systematic review. Conversely, articles not written in English, articles focused on other mycotoxins that did not include the specified keywords, as well as those from outside the geographical scope of Sub-Saharan Africa, were excluded.

### 2.1 Data analysis

The results from the systematic review and network analysis were evaluated to identify the knowledge structure and evolution of masked mycotoxins research in Sub-Saharan Africa. VOSviewer (version 1.6.20) was employed for the network analysis of the selected articles. This software tool analyzes citation data to explore the knowledge structure and evolution of a research field. It was used to create a co-citation network, co-authorship network, and bibliographic coupling to visualize relationships among authors, institutions, keywords, and countries associated with the research.

The analysis focused on the following aspects:

i ***Co-authorship network:*** This network was analyzed to uncover collaboration patterns among researchers and institutions.
ii ***Co-citation network:*** This analysis aimed to identify the core intellectual contributions and the structural connectivity of scholarly outputs involving masked mycotoxin research in Sub-Saharan Africa (SSA)
iii ***Bibliographic coupling:*** This analysis was performed to identify patterns of co-citation and intellectual structures among the retrieved studies, revealing clusters of highly interconnected documents that shared common references.

Additionally, a thematic analysis was conducted to identify key themes and sub-themes within the literature. This analysis was based on the abstracts and full texts of selected articles, using an inductive approach where themes emerged from the data rather than being predetermined. The thematic analysis followed these steps:

i ***Familiarization:*** The abstracts and full texts of the selected articles were reviewed to gain a general understanding of their content.
ii ***Theme identification:*** Key concepts, themes, sub-themes, and ideas that arose from the data were identified.
iii ***Theme refinement:*** The identified themes were refined and validated through an iterative process of analysis and review.

The results from the systematic review, network analysis, and thematic analysis were synthesized to provide a comprehensive understanding of the knowledge structure and evolution of masked mycotoxins research in Sub-Saharan Africa.

## 3 Results and discussion

### 3.1 Description of data

The literature search initially identified a total of 2575 records, categorized by four key search terms: “emerging mycotoxin” (926 records), “modified mycotoxin” (1352 records), “hidden mycotoxin” (71 records), and “masked mycotoxin” (226 records). These records were consolidated into a single Excel.csv file and then filtered based on the research focus and the authors' countries of affiliation, which reduced the dataset to 81 records.

**Fig. 2:**
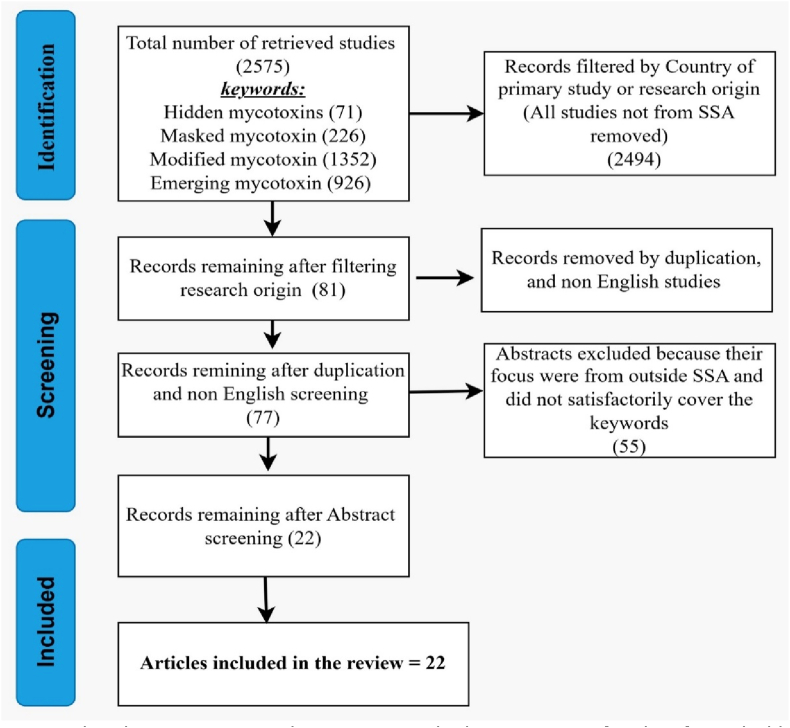
Flowchart summarizing the screening and selection process for identifying eligible studies.

Further refinement included the elimination of duplicates and restrictions on language, resulting in a final dataset of 77 records. The remaining articles underwent a screening process where, guided by predefined inclusion and exclusion criteria, their abstracts were fully read, resulting in the removal 55 studies. After this rigorous selection process, 22 studies were deemed eligible for inclusion (Abass et al., 2019; Adetunji et al., 2014; Agwupuye et al., 2021; Angula et al., 2024; Atnafu et al., 2024; C. Chilaka et al., 2016, 2017, 2018a, 2018b; Ekwomadu et al., 2020; Ezekiel et al., 2013; Getahun et al., 2023; Kemboi et al., 2020a, Kemboi et al., 2020b; Krausová et al., 2022; Maxwell et al., 2018; Mohammed et al., 2022, 2023; Nafuka et al., 2019; Olopade et al., 2021b; Rofiat et al., 2015; Šarkanj et al., 2018; Tebele et al., 2020). All articles considered in this review were published between 2013 and 2024. A detailed overview of the screening process is presented in Fig. 2.

### 3.2 General characteristics of masked mycotoxin research in Sub-Saharan Africa

A total of 22 studies published between 2013 and 2024 were identified in this review as related to masked, hidden, emerging, or modified mycotoxins within the Sub-Saharan African (SSA) context. The bibliometric profile indicates both low research intensity and regional imbalance in the scientific exploration of these compounds.

#### 3.2.1 Trends in research output

**Fig. 3:**
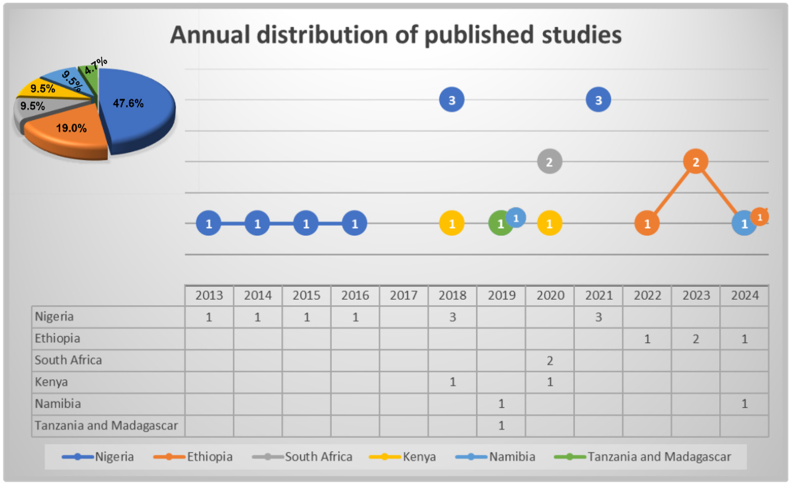
Annual distribution of published studies on masked mycotoxins in Sub-Saharan Africa (SSA) from 2013 to 2024, disaggregated by country.

The earliest identified study was published in 2013, with subsequent years (2014–2017) recording only one publication annually. A relative increase occurred in 2018, during which four studies were published. This upward trend persisted modestly, with two to three publications annually between 2019 and 2024, as shown in Fig. 3 below for visual representation. Despite this gradual growth, the overall volume remains low, suggesting that the topic has not yet achieved widespread recognition in the regional research agenda.

Moreover, analysis of research depth reveals that only three (Ekwomadu et al., 2020; Krausová et al., 2022; Olopade et al., 2021)out of the 22 studies (13.6 %) explicitly focused on masked, hidden, emerging or modified mycotoxins as part of the primary research objectives. The remaining 81.8 % of studies included these compounds merely as part of broader discussions or ancillary findings, indicating limited dedicated attention. This reflects a potentially low level of awareness, analytical capability, or funding support for a comprehensive investigation into these emerging food contaminants.

#### 3.2.2 Geographic distribution of research

A geographical breakdown, as shown in Fig. 3 above, indicates that research efforts are concentrated in a small number of countries, with Nigeria accounting for 10 publications (47.6 %). Other countries contributing more than one study include Ethiopia (n = 4), South Africa (n = 2), Kenya (n = 2), and Namibia (n = 2). One study was co-attributed to Tanzania and Madagascar. This imbalance may reflect disparities in research infrastructure, funding availability, and institutional prioritization of food safety across the region. Nigeria's leading position can be attributed to several structural and institutional factors. The country benefits from relatively higher investment in agricultural research and a more established research ecosystem, including internationally recognized institutions such as the International Institute of Tropical Agriculture (IITA). This major research institution, focused on developing sustainable food production systems in tropical Africa, has historically coordinated mycotoxin surveillance and mitigation initiatives in West Africa (Misihairabgwi et al., 2019; Stepman, 2018).

Furthermore, mycotoxin research in Nigeria began in the early 1960s, spurred by a growing global awareness of aflatoxins and their implications for food safety (Atanda et al., 2013). Initial efforts were led by national institutions such as the Institute for Agricultural Research (IAR) and the Nigerian Stored Products Research Institute (NSPRI), with technical support from the UK's Tropical Products Research Institute (McDonald and AuthorAnonymous, 1969). Nigerian researchers have continued to foster collaborations with international partners, particularly through initiatives like the Pan Africa Chemistry Network and EU-funded programs, both of which have enhanced analytical capabilities and promoted capacity-building efforts (Atanda et al., 2013). Institutions such as IITA have been instrumental in sustaining mycotoxin research and advocacy, despite facing some systemic challenges (Bankole and Adebanjo, 2003). They have also supported sustained scholarly output, contributing to the consistent publication activity observed from 2013 to 2021 in Fig. 3 above. In contrast, countries such as Ethiopia began contributing more recently, from 2022 onward, while South Africa, Kenya, and Namibia exhibited intermittent involvement without a sustained research trajectory. This sporadic engagement highlights the absence of a cohesive, region-wide research strategy on masked mycotoxins.

#### 3.2.3 Systematic reviews and knowledge gaps

Importantly, the review identified no systematic review focused exclusively on masked or modified mycotoxins in the SSA region. Only one review (Chilaka et al., 2017) was found to make a partial reference to modified mycotoxins within a broader discussion on Fusarium. This absence of comprehensive reviews represents a significant gap in the literature, particularly in light of the potential health risks and regulatory implications associated with these compounds. Masked mycotoxins can escape conventional detection methods, posing underappreciated risks to consumers and undermining existing food safety surveillance systems.

These findings underscore the formative nature of masked mycotoxin research in SSA: the field is underexplored, thematically fragmented, and disproportionately concentrated in a few countries. There is a pressing need for investments in analytical capacity, training, inter-country collaboration, and the integration of masked mycotoxin surveillance into national food control systems. Future research agendas should prioritise systematic reviews, risk assessments, and field surveillance studies to support evidence-based policymaking in the region.

### 3.3 Network analysis

#### 3.3.1 Co-authorship network analysis

**Fig. 4:**
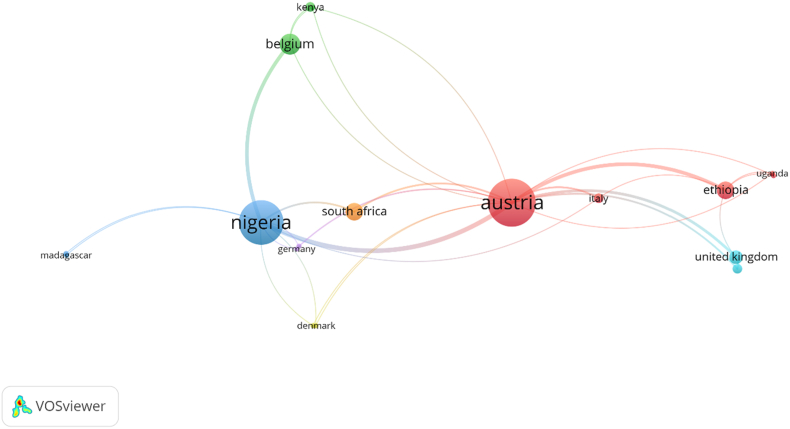
VOSviewer-generated co-authorship network map of countries involved in masked mycotoxin research in Sub-Saharan Africa (2013–2024). Node size represents the number of documents a country is involved in, while link thickness indicates the strength of collaboration.

**Fig. 5:**
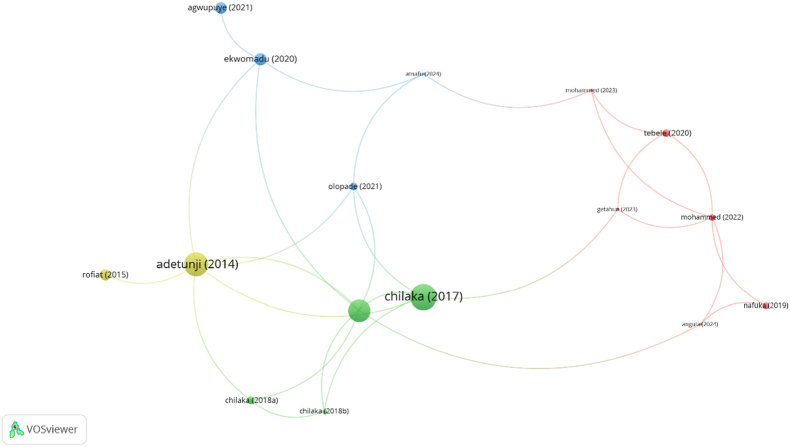
VOSviewer-generated co-citation network map of key documents involving masked mycotoxin research in Sub-Saharan Africa. Node size reflects the total number of citations per document, while link thickness indicates co-citation frequency.

To explore the collaborative landscape in masked mycotoxin research within Sub-Saharan Africa (SSA), a co-authorship analysis was conducted using **VOSviewer**. The results (Fig. 4) show a dispersed and externally supported network, with collaboration nodes predominantly situated outside the continent. Austria emerged as the most central non-African country, with links to 13 publications and a total link strength of 22, followed closely by Nigeria, which was linked to 12 publications and a total link strength of 18. This highlights Nigeria's pivotal role in the region's scientific output and its strong engagement in international consortia. Other key collaborative actors include Ethiopia (link strength = 8), Belgium (7), and the United Kingdom (6), suggesting the presence of well-defined research clusters often driven by North-South academic partnerships. Countries such as Italy, Germany, Denmark, and the United States featured with fewer documents, but relatively high citation counts and strong link strengths, reflecting strategic, high-impact collaborations rather than volume-driven outputs (see Fig. 5).

While these international cooperation models such as Austria's leading role have facilitated access to funding, advanced analytical infrastructure, methodological standardizations and global visibility for African research institutions, they also come with limitations. Such models may perpetuate structural dependencies, constrain agenda-setting autonomy, and risk the marginalization of local research priorities. The dominance of Northern actors underscores the importance of critically evaluating power asymmetries in research partnerships to ensure mutual benefit and long-term sustainability.

The map generated by VOSviewer (Fig. 4) reveals limited South–South collaboration, with few direct links among African countries beyond Nigeria, Ethiopia, and South Africa. Countries like Uganda, Tanzania, Madagascar, and Zambia appeared only peripherally, indicating both underrepresentation and potential entry points for capacity development. These patterns reinforce the conclusion that masked mycotoxin research in SSA is underexplored, and it remains dependent on external collaboration frameworks, with inadequate intra-regional integration. The findings also call for strengthened intra-African scientific alliances and institutional investments to support more autonomous, contextually grounded research on emerging food contaminants.

#### 3.3.2 Citation and co-citation analysis

Citation and co-citation analyses were conducted to identify the core intellectual contributions and the structural connectivity of scholarly outputs involving masked mycotoxin research in Sub-Saharan Africa (SSA). This aligns with the broader objective of the study to uncover how knowledge in this emerging field is disseminated, accumulated, and interlinked through influential studies.

The most cited document was Chilaka et al. (2017) with 100 citations and five co-citation links, followed by Adetunji et al. (2014) with 89 citations and six links, and Chilaka et al. (2016) with 82 citations and seven links, marking them as central nodes in the intellectual structure of this field. These documents, focused on the occurrence and toxicological significance of mycotoxins in food systems, appear repeatedly in the bibliographies of other studies, confirming their role in shaping foundational understanding. The high link strength of Chilaka et al. (2016) suggests that it not only garners citations but is also frequently cited in conjunction with other key works, indicating thematic coherence and sustained academic relevance.

The emerging cluster of recent studies such as Mohammed (2022, 2023), Getahun et al. (2023), Angula et al. (2024), and Atnafu (2024) demonstrates a growing research interest in masked and modified mycotoxins. While their current citation counts and co-citation links (3–5) remain modest, this likely reflects their recent entry into the literature and a limited diffusion within the academic community rather than limited relevance. For instance, Mohammed's work is notable for its methodological rigor, employing LC-MS/MS for multi-mycotoxin detection and for introducing data from underrepresented regions such as Ethiopian maize and sorghum systems. These studies mark an important early effort to quantify post-harvest mycotoxin exposure in East Africa and may signal the initial stages of thematic evolution within the field.

Interestingly, some highly cited documents, such as Kemboi (2020; 48 citations) and Ezekiel et al. (2013; 44 citations) exhibit zero co-citation links. This suggests that although these works are prominent in general mycotoxin research, they may not yet be positioned as central references in the more specialized discourse around masked, modified, or hidden mycotoxins. Conversely, the relatively high co-citation links of works such as Ekwomadu (2019) and Olapade (2021) (both with four links) despite moderate citation counts, point to their relevance in defining conceptual intersections, possibly in analytical techniques or risk assessment frameworks.

The co-citation analysis thus reveals a dual-layered knowledge structure: a foundational layer dominated by a few high-impact works and an emergent layer characterized by newer studies with potential for future centrality. This aligns with the broader trends observed in the review namely, that masked mycotoxin research remains an underdeveloped subfield, with knowledge production anchored in a limited set of seminal contributions and evolving thematic clusters. These findings underscore the need for more targeted research, comprehensive reviews, and regional capacity to generate and cite locally contextualized data. Strengthening these dynamics would enhance the epistemic autonomy of SSA scholars in masked mycotoxin research and support the development of more integrated and representative food safety policies.

#### 3.3.3 Bibliographic coupling analysis

**Fig. 6:**
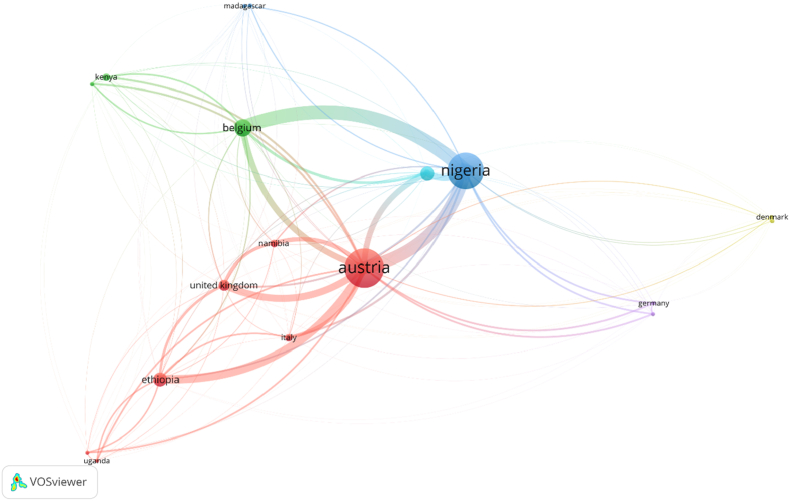
VOSviewer-generated bibliographic coupling map of countries contributing to masked mycotoxin research in Sub- Saharan Africa. Node size indicates the number of documents; link thickness represents the degree of shared references (total link strength). Strong coupling between Austria, Nigeria, and Belgium suggests a shared foundational knowledge base, while more isolated nodes indicate citation divergence.

Bibliographic coupling analysis was performed to assess the degree of shared intellectual basis between countries engaged in research that involved masked mycotoxin in Sub-Saharan Africa (SSA). The analysis, visualized in VOSviewer (Fig. 6 below), provides insights into the thematic and citation-based proximities of national research outputs, reflecting how countries draw upon similar knowledge sources even if they do not collaborate directly.

Austria emerged as the most strongly coupled country, with 13 documents, 432 citations, and an exceptionally high total link strength of 2536, indicating that Austrian researchers' publications share numerous references with those from other countries. This strong coupling suggests Austria's research is highly integrated into the global scientific discourse on studies that involved masked mycotoxins and potentially reflects its role in collaborative multi-country projects involving SSA partners. While Austria exhibited the highest coupling strength, similar though less pronounced intellectual linkages were also observed for Belgium and the United Kingdom, reflecting their engagement in SSA-focused research through shared intellectual networks and targeted funding for mycotoxin research in SSA as well as methodological and policy-oriented contributions, respectively.

Nigeria, with 12 documents and a total link strength of 1969, ranked second, reaffirming its central role not only in co-authorship (as discussed earlier) but also in conceptual and referential alignment with broader research efforts. The high coupling strength signifies Nigeria's publications frequently cite similar foundational literature as those from Austria, Belgium, Ethiopia, and others, evidence of Nigeria's embeddedness within the intellectual fabric of the field. Other notable countries include Belgium (1245), Ethiopia (848), and the United Kingdom (777). Their bibliographic coupling strengths reflect significant overlaps in cited literature, particularly in studies addressing food safety, mycotoxin occurrence, and detection methods. Meanwhile, Namibia (494) and South Africa (752) also demonstrated moderate coupling, suggesting an emerging alignment with the global literature base, despite their relatively lower publication volume.

Interestingly, countries such as Germany and the United States presented high citation counts (107 each) and moderate coupling strengths (235), indicating their outputs are well-regarded yet perhaps less frequently cite the same core sources as other SSA-associated research groups. This may signal epistemic divergence or context-specific citation practices.

The bibliographic coupling network supports the broader theme of this study: masked mycotoxin research in SSA is being built upon shared global knowledge bases, yet there is clear variability in the depth and nature of conceptual interlinkages across countries. These findings emphasize the importance of harmonizing citation practices and promoting regionally cohesive literature development, critical steps for strengthening the intellectual cohesion of SSA's research on masked mycotoxins.

### 3.4 Prevalence and distribution of masked mycotoxins in Sub-Saharan African crops

Several studies have documented the prevalence of mycotoxins in staple crops across SSA. These studies often focus on major mycotoxins like aflatoxins, fumonisins, zearalenone, and trichothecenes (Chilaka et al., 2017; Kemboi, Antonissen, et al., 2020; Nleya et al., 2018). However, the presence of masked mycotoxins, which are often overlooked in standard analyses due to their modified chemical structures, remains largely unexplored. Research on masked mycotoxins, specifically those derived from Fusarium species such as deoxynivalenol-3-glucoside (DON-3-Glc), is scarce (Gratz, 2017; Nakagawa, 2016). Nevertheless, a few direct studies have begun to shed light on this issue in the SSA context. For example, a study by Getahun et al. (2023) in Ethiopia detected DON-3-glucoside in 64 % of wheat samples, with a maximum concentration of 2120 μg/kg. Other studies by Ekwomadu et al. (2020, 2021) from South Africa identified a spectrum of masked Fusarium metabolites in maize, including DON-3-Glc, zearalenone-14-glucoside, and others. This highlights the need for more comprehensive mycotoxin analysis in SSA, incorporating methods capable of detecting masked mycotoxins. The lack of standardised testing and widespread monitoring programs hinders a complete understanding of the prevalence of these masked mycotoxins across different crops and regions within SSA (Nleya et al., 2018). For example, while some studies have identified high levels of aflatoxins in maize (Aasa et al., 2022; Somda et al., 2023), the co-occurrence and levels of masked aflatoxin metabolites remain largely uninvestigated. This section will analyze the prevalence and distribution of masked mycotoxins across different staple crops in Sub-Saharan Africa.

#### 3.4.1 Maize: prevalence of masked mycotoxins and associated health risks

Maize is a dietary staple across many Sub-Saharan African nations and a major source of masked mycotoxin contamination in the region (Gnonlonfin et al., 2019). Studies consistently demonstrate high levels of mycotoxin contamination in maize, encompassing both free and masked forms(Akello et al., 2021; Ekwomadu et al., 2020). A growing body of research has identified the occurrence of masked mycotoxins in maize from various regions of South Africa (Ekwomadu et al., 2020, 2021) providing valuable insights into the extent and nature of contamination. Specifically, Ekwomadu et al. (2020) conducted a robust multi-regional survey of maize samples using LC-MS/MS, detecting a complex profile of regulated and masked Fusarium metabolites. Their study reported not only dominant levels of fumonisins (B1–B4, A1) but also masked forms such as DON-3-glucoside and zearalenone-14-glucoside. The direct detection of these masked compounds in maize confirms their occurrence in the SSA food system and underscores their toxicological relevance**.** The study highlighted significant differences in contamination patterns across various agricultural regions and maize types.

The high prevalence of fumonisins in maize is particularly concerning given their known toxic effects, which include liver and kidney damage (Ekwomadu et al., 2021). The presence of masked mycotoxins adds another layer of complexity to the problem, as these forms are not readily detected by standard analytical methods (Goryacheva and De Saeger, 2012), potentially leading to underestimation of the true mycotoxin burden. Further research is needed to elucidate the contribution of masked mycotoxins to the overall toxicity of contaminated maize.

#### 3.4.2 Sorghum and millet: prevalence of masked mycotoxins and associated health risks

Sorghum is a key staple cereal in many sub-Saharan African countries, where it contributes substantially to household food security, nutrition, and livelihoods (Ssepuuya et al., 2018). Despite its agronomic resilience and socioeconomic importance, sorghum is highly susceptible to fungal contamination, particularly by Fusarium species, which produce a broad spectrum of mycotoxins, including both regulated and non-regulated forms. Of particular concern are masked, modified, and emerging mycotoxins, which are often under-reported due to limitations in conventional analytical techniques. In a targeted study conducted by Chilaka et al. (2016), the occurrence of hidden fumonisins in cereals—including sorghum, millet, maize and the fermented cereal product ogi was evaluated through a combination of free and hydrolyzed fumonisin quantification. The researchers employed liquid chromatography-tandem mass spectrometry (LC-MS/MS) to detect both free fumonisins (FB_1_, FB_2_, and FB_3_) and their hidden forms released through alkaline hydrolysis. Their analysis revealed the presence of several emerging and modified mycotoxins in the samples, and of particular interest was the presence of 15-acetyl-deoxynivalenol (15-ADON), deoxynivalenol-3-glucoside (DON-3G), zearalenone-14-glucoside (ZEN-14G), α-zearalenol (α-ZEL), and β-zearalenol (β-ZEL) in sorghum and millet samples. These findings constitute some of the most direct and detailed evidence of masked mycotoxins in non-maize cereals in SSA.

The detection of these non-conventional mycotoxins highlights a significant but often overlooked dimension of food safety risk associated with the consumption of sorghum and millet. Given the potential toxicological effects and the absence of regulatory limits for many of these compounds, there is an urgent need to integrate routine multi-mycotoxin surveillance into national and regional food control frameworks. Furthermore, food safety regulations must evolve to reflect the expanding spectrum of mycotoxins relevant to staple crops in sub-Saharan Africa.

#### 3.4.3 Wheat: prevalence of masked mycotoxins and associated health risks

While not as universally prevalent a staple as maize or sorghum across all Sub-Saharan African nations, wheat cultivation and consumption are significant, particularly in Ethiopia (Getahun et al., 2023). Research focusing on Ethiopian wheat grains has revealed substantial multi-mycotoxin contamination, including notable levels of masked mycotoxins. A study by Getahun et al. (2023) analyzed 178 wheat grain samples collected during the 2020 cropping season in Ethiopia. Their findings indicated the presence of various fungal genera, with Fusarium species being particularly abundant. Multi-mycotoxin analysis using LC-MS/MS identified 49 metabolites, categorized into eight groups, encompassing masked, regulated, and emerging mycotoxins (Getahun et al., 2023). Deoxynivalenol (DON) was the most frequently detected mycotoxin, present in approximately 70.8 % of samples, with a maximum concentration of 15,900 μg/kg. Its masked metabolite, DON-3-glucoside, was detected in 64 % of samples, with a maximum level of 2120 μg/kg. This study presents further evidence of masked mycotoxin prevalence in SSA food systems, providing empirical data that directly supports the need for broader inclusion of masked toxins in surveillance frameworks**.** The prevalence and concentrations of these masked mycotoxins highlight a significant food safety and public health risks associated with wheat consumption.

#### 3.4.4 Other crops and research gaps

A comprehensive review by Chilaka et al. (2017) titled “The Status of Fusarium Mycotoxins in Sub-Saharan Africa: A Review of Emerging Trends and Post-Harvest Mitigation Strategies Towards Food Control”, documented the occurrence and contamination levels of emerging and modified Fusarium mycotoxins in food crops and processed products across sub-Saharan Africa from the year 2000 onward. The review provides valuable insights into contamination patterns in multiple countries, including Burkina Faso, Cameroon, Ethiopia, Kenya, Malawi, Mozambique, Nigeria, and Zimbabwe. Various crops were assessed, and their corresponding emerging mycotoxins such as moniliformin (MON), enniatins (ENNs), and fusaproliferin (FUS) were reported. However, the authors highlight a significant regulatory and research limitation: despite the documented presence of modified mycotoxins in food and feed products, establishing regulatory standards remains infeasible due to a persistent lack of comprehensive exposure data and toxicological risk assessments.

The fragmented nature of current studies has hindered substantive progress in elucidating the toxicokinetics and health implications of these modified compounds. Moreover, while some foundational research data on masked mycotoxins exist for key staple crops like maize and sorghum, information regarding their presence in other widely consumed sub-Saharan African crops such as rice, legumes, and cassava remain poorly understood and under-documented. This represents a critical knowledge gap, particularly in the context of food security and public health. There is a pressing need for systematic and regionally representative research to characterize the prevalence of masked mycotoxins in under-investigated staple crops. Such investigations should be underpinned by the use of advanced analytical platforms particularly liquid chromatography coupled with tandem mass spectrometry (LC-MS/MS) which can sensitively detect both free and conjugated toxins forms. Additionally, future research must account for the region's heterogeneous agroecological conditions and post-harvest practices, both of which can influence mycotoxin biosynthesis and transformation. Addressing this research gap is vital for constructing comprehensive risk assessment frameworks and informing the development of regulatory policies aimed at mitigating dietary mycotoxin exposure in vulnerable populations.

### 3.5 Types of masked mycotoxins mostly detected in sub-saharan Africa

Despite the potential health risks, the occurrence, metabolism, and toxicity of masked mycotoxins their presence and research in African crops remain poorly understood. The most frequently detected mycotoxins and their conjugated forms in SSA region are Deoxynivalenol (DON) and its Glucosides and Zearalenone and its Glucosides which have been studied to a greater degree above other forms of masked mycotoxins in the region.

Deoxynivalenol (DON) and its conjugated forms, particularly DON-3-glucoside, are frequently reported in Sub-Saharan African crops (Ekwomadu et al., 2020; Getahun et al., 2023; Ssepuuya et al., 2018). DON-3-glucoside is a plant-derived conjugate formed as a defense mechanism against DON accumulation (Berthiller et al., 2013; Cirlini et al., 2013). However, this masked form can be hydrolyzed in the gastrointestinal tract, releasing free DON and potentially increasing the overall toxic burden (Berthiller et al., 2013). Studies investigating the metabolism of DON-3-glucoside in pigs (Nagl et al., 2014) and rats (Nagl et al., 2012) have shown significant hydrolysis in the intestinal tract. The resulting DON can then be absorbed and contribute to systemic toxicity (Nagl et al., 2012, 2014). In vitro studies using human gut microbiota have also confirmed the efficient hydrolysis of DON-3-glucoside (Daud et al., 2020; Gratz et al., 2017, 2018). The extent of hydrolysis and the subsequent release of DON can vary depending on individual factors and the composition of the gut microbiota (Gratz, 2017). The presence of DON-3-glucoside highlights the importance of considering masked mycotoxins in risk assessments and regulatory limits for food safety.

Zearalenone (ZEN) and its conjugated forms (e.g., ZEN-14-glucoside) have also been detected in Sub-Saharan African crops (Ekwomadu et al., 2020; Hudu et al., 2024). The study by Ekwomadu et al. (2021) found ZEN in a considerable proportion of maize samples from South Africa. ZEN is an estrogenic mycotoxin that can disrupt the endocrine system, particularly in animals. Its masked forms, like ZEN-14-glucoside, may also have estrogenic effects or be hydrolyzed to release free ZEN, however, the metabolism and toxicity of ZEN glucosides are less well-understood than those of DON glucosides, requiring further investigation (Faisal et al., 2019; Righetti et al., 2017). The detection of ZEN and its glucosides in Sub-Saharan African crops necessitates further research to assess their potential health impacts.

### 3.6 Other masked mycotoxins: emerging concerns

Other masked mycotoxins, including those derived from trichothecenes like T-2 toxin and HT-2 toxin (De Boevre et al., 2014; Daud et al., 2020; McCormick et al., 2015), may be present in Sub-Saharan African crops but are less frequently investigated. The study by McCormick et al. (2015) focused on T-2 toxin-glucoside, demonstrating its hydrolysis under conditions simulating human digestion. De Boevre et al. (2014) developed an in vitro model for masked mycotoxin biosynthesis, identifying and characterizing various glucoside derivatives of T-2 toxin and HT-2 toxin. Daud et al. (2020) investigated the ability of various human gut bacteria to hydrolyze mycotoxin glucosides, including those of T-2 toxin and HT-2 toxin. The limited research highlights a critical knowledge gap, as the occurrence and toxicological significance of these masked forms remain largely unknown (De Boevre et al., 2014; Nakagawa, 2016). Further research is essential to identify and characterize the full range of masked mycotoxins present in Sub-Saharan African crops and to assess their potential health risks.

### 3.7 Analytical methods for detecting masked mycotoxins

Accurate detection of masked mycotoxins requires advanced analytical techniques, primarily liquid chromatography-mass spectrometry (LC-MS/MS), which enable the identification and quantification of both free and conjugated mycotoxins (Ekwomadu et al., 2020; Getahun et al., 2023; Ssepuuya et al., 2018). The development of sensitive and specific liquid chromatography coupled with high-resolution mass spectrometry (LC-HRMS) methods has been instrumental in advancing research on masked mycotoxins, especially in developed countries (Lapris et al., 2024) Significant strides in optimising LC-MS/MS protocols for detecting a broad spectrum of masked compounds, including glucosylated, acetylated, and sulfate-conjugated derivatives of DON, ZEN, and other *Fusarium* toxins, have been made across Europe (Boevre et al., 2015; González-Jartín et al., 2019).

These multi-mycotoxin platforms developed in Europe have further improved throughput and matrix compatibility, facilitating global surveillance initiatives. However, access to such advanced equipment and expertise remains limited in many sub-Saharan African countries, hindering comprehensive mycotoxin monitoring and surveillance efforts. The high cost and technical expertise required for LC-MS/MS analysis pose significant challenges for widespread application in resource-limited settings. Immunochemical methods, such as enzyme-linked immunosorbent assays (ELISAs)(Goryacheva and De Saeger, 2012; Saleh et al., 2017), offer a potentially simpler and more cost-effective alternative for screening purposes. The limited access to sophisticated analytical techniques in many sub-Saharan African countries underscores the need to develop and implement affordable and accessible methods for mycotoxin detection and monitoring.

### 3.8 Co-occurrence of mycotoxins: synergistic effects

The simultaneous presence of multiple mycotoxins in food is common (Ekwomadu et al., 2020; Getahun et al., 2023). The combined effects of these co-occurring mycotoxins can be greater than the sum of their individual effects, leading to synergistic toxicity and increased health risks (Gratz, 2017). The study by Kovalsky et al. (2016b) highlighted the frequent co-occurrence of regulated, masked, and emerging mycotoxins in animal feed and maize. The complex interactions between multiple mycotoxins and their masked forms require further investigation to fully understand their combined health effects, particularly in Sub-Saharan Africa where multi-contamination is frequently observed (Ekwomadu et al., 2020; Getahun et al., 2023). This understanding is crucial for developing effective risk assessment and management strategies.

### 3.9 Research gaps and future directions

#### 3.9.1 Improved mycotoxin monitoring and surveillance

Comprehensive mycotoxin monitoring and surveillance programs are crucial for assessing the extent of contamination and guiding effective interventions (Wild et al., 2015). This requires access to advanced analytical methods, trained personnel, and robust data management systems. Studies should focus on identifying high-risk areas and populations, characterizing the types and levels of mycotoxins present, and monitoring the effectiveness of mitigation strategies. The development of rapid, cost-effective screening methods suitable for use in resource-limited settings is also essential (Wild et al., 2015). Investments in infrastructure, training, and capacity building are crucial for establishing effective mycotoxin monitoring and surveillance systems.

#### 3.9.2 Understanding the metabolism and toxicity of masked mycotoxins

Further research is needed to fully understand the metabolism and toxicity of masked mycotoxins (Gratz, 2017; S. W. Gratz et al., 2017). Studies should investigate the extent of hydrolysis of masked mycotoxins, their absorption and distribution, and their potential for synergistic interactions with other mycotoxins. This knowledge is crucial for accurate risk assessment and the development of effective management strategies. Studies should also investigate the long-term health consequences of chronic exposure to masked mycotoxins.

#### 3.9.3 Development of novel mitigation strategies

The development of novel and sustainable mitigation strategies is essential to address the problem of mycotoxin contamination (Monda and Alakonya, 2016). This includes the breeding of crop varieties with enhanced resistance to fungal infection, the development of improved post-harvest technologies, and the exploration of innovative decontamination methods. These strategies should be tailored to the specific contexts of Sub-Saharan African countries, considering the local climate, farming practices, and resource availability. Further research should focus on developing cost-effective and sustainable solutions that are accessible to smallholder farmers.

## 4 Conclusion

This systematic review and network analysis reveals a nascent but increasingly relevant body of research on masked mycotoxins in Sub-Saharan Africa (SSA). The findings underscore that Deoxynivalenol (DON) and Zearalenone (ZEN) conjugates are the most commonly studied, although the region's broader mycotoxin landscape remains critically underexplored. Most studies treat masked mycotoxins as secondary findings rather than primary targets, reflecting limited prioritization in both research design and policy discourse.

The geographic concentration of studies in Nigeria, with sparse contributions from other SSA countries, highlights an uneven research landscape. This limitation, coupled with inadequate analytical infrastructure, has constrained the region's capacity to detect, quantify, and regulate masked mycotoxins. Particularly concerning is the frequent co-occurrence of masked, free, and emerging mycotoxins in staple crops like maize and sorghum, posing unquantified risks to food safety, especially among vulnerable populations such as children and women of reproductive age.

To address these challenges, SSA must invest in upgrading detection capabilities, including access to LC-MS/MS technologies and the development of cost-effective, field-level diagnostic tools. There is also a need for targeted toxicological research that considers local diets and gut microbiota, which influence mycotoxin metabolism.

Ultimately, a coordinated, multi-sectoral response is needed encompassing surveillance systems, public awareness campaigns, regulatory reform, and strengthened intra-regional collaboration. Integrating masked mycotoxins into national food safety and health frameworks will be vital to safeguard public health and enhance regional resilience against mycotoxin-related risks.

## CRediT authorship contribution statement

**Chimwemwe Chilenga:** Writing – original draft, Visualization, Methodology, Formal analysis, Data curation, Conceptualization. **William Kasapila:** Writing – review & editing, Validation, Supervision. **Kingsley Masamba:** Validation, Supervision, Data curation. **Tinnah Manani:** Supervision, Conceptualization. **Victor Munkhuwa:** Writing – review & editing, Data curation. **Brown Ndhlovu:** Formal analysis, Data curation. **Kennedy Machira:** Writing – review & editing, Validation, Supervision, Data curation.

## Ethical statement

We, the authors of the manuscript titled **“Knowledge Structure and Evolution of Masked Mycotoxin Research in Sub-Saharan Africa: A Systematic Review and Network Analysis Approach,”** affirm that the work submitted is original, has not been published previously, and is not under consideration for publication elsewhere.

As this is a systematic review and network analysis based entirely on previously published studies, the research did not involve any human participants, animal subjects, or identifiable personal data, and therefore, ethical approval and informed consent were not required.

All data used in this review were obtained from publicly accessible sources, and the study was conducted in accordance with recognized ethical standards for secondary research. Proper citation and acknowledgment of all referenced sources have been ensured.

All authors have reviewed and approved the final manuscript and agree to its submission. There are no conflicts of interest to declare.

## Declaration of competing interest

The authors declare that they have no known competing financial interests or personal relationships that could have appeared to influence the work reported in this paper.

## Data Availability

No data was used for the research described in the article.
